# Molecular Epidemiological Investigation of *Cyclospora* spp. in Holstein Cattle in Partial Areas of the Yunnan Province, China

**DOI:** 10.3390/ani13091527

**Published:** 2023-05-03

**Authors:** Jian-Fa Yang, Zhao-Jun Heng, Fan-Fan Shu, Hua-Ming Mao, Yong-Sheng Su, Jun-Jun He, Feng-Cai Zou

**Affiliations:** 1 Key Laboratory of Veterinary Public Health of Yunnan Province, College of Veterinary Medicine, Yunnan Agricultural University, Kunming 650201, China; jsc315@163.com (J.-F.Y.); hzhaoj123@126.com (Z.-J.H.); shuff1227@163.com (F.-F.S.); 2 Key Laboratory of Animal Nutrition and Feed in Yunnan Province, College of Animal Science and Technology, Yunnan Agricultural University, Kunming 650201, China; 1985039@ynau.edu.cn; 3 Yunnan New Hope Xuelan Animal Husbandry Technology Co., Ltd., Qujing 650201, China; 15887136661@163.com

**Keywords:** *Cyclospora* spp., Holstein cattle, infection rate, molecular identification

## Abstract

**Simple Summary:**

*Cyclospora* spp. is an important zoonotic parasite that poses a threat to public health. At present, the research on *Cyclospora* spp. in cattle of the Yunnan Province remains limited. We firstly reported the prevalence of *Cyclospora* spp. in Holstein cattle in the Yunnan Province, China. In order to understand the prevalence and genotype of *Cyclospora* spp. in the Yunnan Province, 524 fecal samples from 4 regions were collected and tested. The results of the present study showed that 13 samples were positive for *Cyclospora* spp., and the total infection rate of *Cyclospora* spp. was 2.48%. We analyzed different risk factors, such as regions, sexes, and ages, but the difference was not statistically significant. Phylogenetic analysis showed that five *Cyclospora* spp. samples were classified into the zoonotic group of *Cyclospora cayetanensis*, which had zoonotic potential. The results of the present study indicated that *Cyclospora* spp. in the Holstein cattle of Yunnan Province posed a risk of zoonosis.

**Abstract:**

*Cyclospora* spp. is a food-borne intestinal protozoan, which is widely distributed in the world and poses the risk of zoonosis. In order to reveal the prevalence of *Cyclospora* spp. in Holstein cattle in partial areas of the Yunnan Province, 524 fresh fecal samples of Holstein cattle were collected from Dali, Kunming, Chuxiong, and Qujing in Yunnan Province. A nested PCR amplification of the small subunit (SSU) rRNA gene of *Cyclospora* spp. was carried out, and the products of the nested PCR were further analyzed by restriction fragment length polymorphism (RFLP) using *Bsp E* Ⅰ. The results of the present study showed that 13 samples were positive for *Cyclospora* spp., and the total infection rate of *Cyclospora* sp. was 2.48%. The infection of *Cyclospora* spp. was detected in Dali, Qujing, and Chuxiong. Chuxiong showed the highest infection rate (5.71%), and infection rate in Dali and Qujing was 2.19% and 3.16%, respectively. Interestingly, the infection of *Cyclospora* spp. was not detected in Kunming. The infection of *Cyclospora* spp. showed no significant differences among different regions (*p* > 0.05). *Cyclospora* sp. infection was detected in all ages and sexes, but the differences were not significant (*p* > 0.05). Sequencing and phylogenetic analysis showed that five *Cyclospora* spp. samples were closely related to the *Cyclospora* spp. of humans, and the others were closely related to the *Cyclospora* spp. of bovines. The results of the present study suggested that there was an infection of *Cyclospora* spp. in Holstein cattle in the Yunnan Province, and the *Cyclospora* spp. showed a risk of zoonosis. Thus, the prevention and control of *Cyclospora* spp. should be strengthened in the Yunnan Province, China. The results of this investigation provide data references for the further research of Cyclosporiasis in Holstein cattle in the Yunnan Province.

## 1. Introduction

*Cyclospora* spp. belongs to the protozoa subkingdom, subphylum Apicomplexa, subclass Coccidiasina, order Eucoccidiorida, family Eimeriidae, and *Cyclospora* [1]. *Cyclospora* spp. is a food-borne zoonotic intestinal protozoan that is widely distributed in the world. It is mainly transmitted through contaminated water or food, and it can also be transmitted through direct contact between humans and animals [2]. *Cyclospora* spp. mainly infects the epithelial cells of the upper part of the small intestine, especially the jejunum [1]. The clinical symptoms of *Cyclospora* spp. infection depend on the host’s immunity, and the symptoms of infection include watery diarrhea, nausea, abdominal cramps, weight loss, and even death in severe cases [3,4]. *Cyclospora* spp. is an opportunistic pathogenic protozoan, and the infection of *Cyclospora* spp. in normal immunity shows inapparent infection or self-limited diarrhea. However, individuals with immune deficiencies can have persistent diarrhea [5], dehydration diarrhea, and even death in severe cases [6]. There are a large number of oocysts in the feces of patients infected with *Cyclospora* spp. Thus, feces are an important source of infection. The oocysts of *Cyclospora* develop into infectious oocysts in the environment, and humans are infected via ingesting water and food contaminated by oocysts [7]. Oocysts enter the host and invade the epithelial cells of host’s small intestine where produces merozoites and oocysts [8]. *Cyclospora* spp. and *Eimeria* also have a high similarity in morphology, and *Cyclospora* spp. was once thought to be a cyanobacterium-like or coccidian-like body (CLB) [9]. With the increase in research, *Cyclospora* spp. has been clearly classified.

*Cyclospora* spp. is an emerging pathogen causing worldwide outbreaks of cyclosporiasis. *Cyclospora* spp. can be found in a wide range of animals, including the Diplopoda, Reptilia, and Insectivora classes, as well as the mammalian orders Rodentia and Primate [10]. Up to now, 20 species of *Cyclospora* spp. have been reported [2,4,11]. Although most of these *Cyclospora* spp. species are host-specific, *Cyclospora cayetanensis* (*C. cayetanensis*) has been currently confirmed as zoonotic pathogen, and it is the only known *Cyclospora* species that can infect humans [8]. Humans are thought to be infected by *C. cayetanensis* through ingesting food or water contaminated with oocysts [12]. Environmental factors such as contaminated fruits, vegetables, water, and soil are considered as the main sources of *C. cayetanensis* infection in humans [13]. *C. cayetanensis* DNA can be detected in the fecal samples of various animal species [14], but there are no data to confirm whether animals are involved in the spread of *C. cayetanensis* [7], and it is generally believed that animals act as mechanical carriers of oocysts.

*Cyclospora* spp. is an emerging zoonotic intestinal protozoan that poses a threat to human health. Since the discovery of *Cyclospora* spp., a large number of studies have been conducted to reveal the morphology and molecular characters of *Cyclospora* spp. in various vertebrates. The oocysts of *C.cayetanensis* have been identified in the feces of human infants, pupils, and adults in the Anhui Province by using microscopes, and minor sequence polymorphisms were observed in the 18S rRNA gene of *C.cayetanensis* in the Henan province, China. Three new *Cyclospora* species from monkeys were identified in Ethiopia by using morphology and molecular characterizations. The 18S rRNA gene fragments of *Cyclospora*-like organisms have also been detected from non-human primates in China [15,16,17,18,19]. *Cyclospora*-like oocysts that belong to the group of primate-derived *Cyclospora* spp. have been found in the fecal specimens of cattle in China [20]. The Yunnan Province is located in the southwest frontier of China and covers an area of 394,100 square kilometers. Yunnan has diverse climates and is suitable for the growth of all kinds of high-quality forage grass, which contribute to the rapid development of dairy farming in Yunnan. Holstein cattle are farmed on a large scale, and parasitic infections may occur during the breeding process. *Cyclospora* spp. not only affects the development of the farming industry, but it is also a potential threat to the health of humans. However, there is a lack of relevant information about the infection of *Cyclospora* spp. in Holstein cattle in Yunnan.

In this study, 524 Holstein cattle fecal samples were randomly collected from different Holstein cattle farms in Dali, Kunming, Chuxiong, and Qujing in the Yunnan Province, and all of these collected samples were used for *Cyclospora* spp. detection. The nested PCR amplification was performed by using primers designed according to the 18S rRNA sequence of the *Cyclospora* spp. A restriction endonuclease fragment length polymorphism (RFLP) analysis with *Bsp E* I was used to differentiate the *Cyclospora* species from cattle *Eimeria* spp. In this study, the epidemic situation of *Cyclospora* spp. was investigated, its distribution was clarified, and the risk of zoonotic transmission of *Cyclospora* spp. in Holstein cattle in the Yunnan Province was also evaluated. At last, the phylogenetic tree was constructed. The results of the present study provide important epidemic data that could support the prevention or control of cyclosporiasis in Holstein cattle in the Yunnan Province.

## 2. Materials and Methods

### 2.1. Specimens

In total, 524 fecal samples were collected from Holstein farms in Dali, Kunming, Qujing, and Chuxiong in the Yunnan Province from July to November 2021. The ages of the Holstein cattle ranged from newborn calves to adult cattle. Among them, there were 422 female Holstein cattle feces samples and 102 male Holstein cattle feces samples. Feces samples were collected directly from the rectum using disposable gloves, separately transferred into disposable plastic bags, recorded with their collection date, ages, and geographic information, and stored at 4 °C until used for DNA extraction.

### 2.2. DNA Extraction and PCR

The fecal sample was added to the beaker, washed with distilled water, filtered, centrifuged at 3000 r/min for 3 min, the supernatant was discarded, and 250 µL of the precipitate was aspirated into a 2 mL centrifuge tube and used for DNA extraction. The DNA of all samples were extracted individually by using the E.Z.N.A.R^®^ Stool DNA Kit (Omega Bio-tek Inc., Norcross, GA, USA), and the extracted DNA were stored at −20 °C until use. *Cyclospora* spp. in the specimens were genetically characterized by nested PCR amplification of a 501 bp fragment of the small subunit (SSU) rRNA gene. Primers for the fragment (501 bp) were designed as previously described [20]. 

The primers used for the primary nested amplification of the SSU gene were 18S-F1 (5′-AATGTAAAACCCTTCCAGAGTAAC-3′) and 18S-R1 (5′-GCAATAATCTATCCCCATCACG-3′), 18S-F2 (5′-AATTCCAGCTCCAATAGTGTAT-3′), and 18S-R2 (5′-CAGGAGAAGCCAAGGTAGGCRTTT-3′) were used for secondary PCR amplification.

PCR was conducted in a 25 µL reaction system containing 10× PCR buffer, 200 µM dNTP, 0.4 µM of each primer, 1 unit of TaKaRa Ex-Taq DNA polymerase (TaKaRa Co., Ltd., Tokyo, Japan), and a DNA sample. In the first round of the PCR reaction system, 1 µL of DNA sample was added, but two microliters of primary PCR product were used as the template for the secondary nested PCR. The primary amplification contained 35 cycles (95 °C for 45 s, 55 °C for 45 s and 72 °C for 1 min), with an initial hot start at 94 °C for 7 min and a final extension at 72 °C for 10 min. The secondary cycling parameters were identical to the first-round amplifications with modified extension time, which was increased to 1 min 30 s. After the amplification complete, electrophoresis was performed on 1% agarose gel under 120 V for 30 min. The results of the electrophoresis were photographed in an ultraviolet gel imaging device.

### 2.3. Restriction Fragment Length Polymorphism (RFLP) Analysis

Positive nested PCR products were further analyzed by RFLP to differentiate *Cyclospora* spp. from *Eimeria* spp. The RFLP reaction system contained 1 U volume of restriction endonuclease *Bsp E* I (10,000 U/mL), 12.5 μL of secondary amplification products, 2.5 μL of 10× NE Buffer 3.1, and 10 μL ddH_2_O. The samples were incubated at 37 °C for 4–8 h. Then, the products of the enzyme digestion were mixed with 1 µL of 10× loading buffer and separated by using electrophoresis on a 2% agarose gel. The results of electrophoresis were photographed by using a gel imaging system.

### 2.4. Sequence Analysis and Phylogenetic Analysis

The PCR products that were successfully digested by *Bsp E* I were sent to Shenggong Bioengineering Co., Ltd. (Shanghai, China) for Bi-directional sequencing. The sequences obtained in this study were further searched for homologous sequences using the GenBank BLAST service. The homologous sequences were downloaded and used as reference data. The sequence alignment analyses were conducted by using the software MEGA 7. The neighbor-joining tree based on sequences of the SSU rRNA gene was constructed using genetic distances of Kimura 2-parameter model. Bootstrap analysis was conducted using 1000 replicates. In this study, those with a node support value higher than 50% were displayed, and the bootstrap analysis was considered significant when the support value greater than 95%. The SSU rRNA gene sequences of *Cyclospora* spp. obtained in this study had been submitted to GenBank and can be accessed using accession numbers OP268222-OP268234.

### 2.5. Statistical Analysis

The χ^2^ test was applied to analyze the differences in the infection rate of *Cyclospora* spp. in Holstein cattle among different regions, different ages, and different genders. The confidence interval was set as 95%, and *p* < 0.05 was used as threshold of statistical significance. OR > 1 indicated that the factor is a risk factor; OR < 1 indicated that the factor is a protective factor. The lower limit of 95% CI greater than 1 indicates that this factor is a risk factor; an upper limit < 1 indicated that this factor is a protective factor. All statistical analyses were performed by using the statistical software SPSS 20.0.

## 3. Results

### 3.1. PCR Amplification and RFLP Analysis

**Figure 1:**
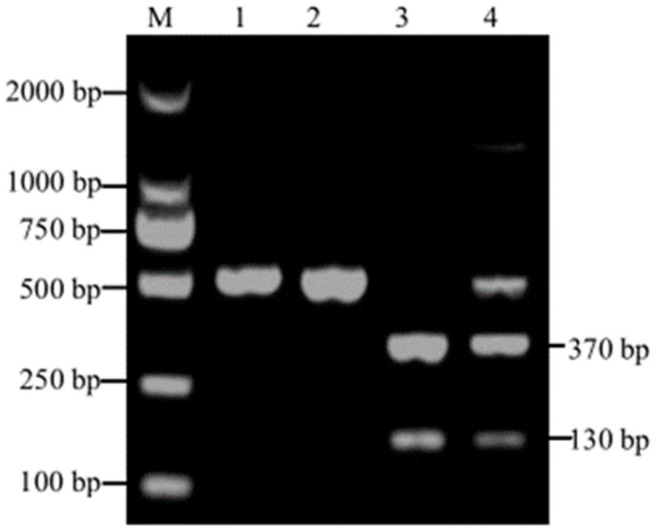
Partial SSU rRNA gene amplicons digested with restriction endonuclease *Bsp E* I. Lane M: DL 2000 DNA Marker; lanes 1–4: *Bsp E* I digested products from nested PCR. The original Western Blot Figure is Figure S1.

In this study, the amplified DNA fragments of *Cyclospora* spp. and *Eimeria* spp. were both 500 bp. To distinguish *Cyclospora* spp. from *Eimeria* spp., RFLP analysis was subsequently performed on these PCR amplicons using the restriction endonuclease *Bsp E* I. The PCR amplicon of *Cyclospora* spp. SSU rRNA gene can be digested into 130 bp and 370 bp fragments, but the PCR product amplified from *Eimeria* spp. can not be digested. In this study, the PCR products amplified from 13 samples were digested into two fragments (130 and 370 bp), and the global positive ratio of *Cyclospora* spp. was 2.48% (13/524) (Table 1). As shown in Figure 1, the PCR amplicons from *Eimeria* spp. were not digested (Figure 1, lanes 1 and 2), whereas PCR amplicons from the *Cyclospora* spp. had been digested into two fragments (130 and 370 bp) (Figure 1, lane 3). One PCR amplicon showed three fragments (130, 370, and 500 bp) (Figure 1, lane 4). It might be the result of the co-infection of *Eimeria* spp. and *Cyclospora* spp. A total of three samples showed co-occurrences of three bands.

### 3.2. Risk Factors of Cyclospora spp. Infection

In this study, the Holstein cattle samples were collected from Dali, Kunming, Qujing, and Chuxiong in Yunnan Province. Although *Cyclospora* spp. infection was not detected in Kunming, the prevalence of *Cyclospora* spp. was found in the other three places, among which Chuxiong had the highest infection rate of 5.71% (2/35), followed by Qujing (3.16%, 5/158) and Dali (2.19%, 6/274). There was no significant difference among the four regions (*p* > 0.05) (Table 1).

In total, 524 samples were divided into four age groups, including 0–3 months, 3–6 months, 6–12 months, and more than one year. The infection of *Cyclospora* spp. was found in all age groups, and the infection rate in the 0–3 months, 3–6 months, 6–12 months, and more-than-one-year groups was 1.74%, 5.26%, 9.09%, and 2.68%, respectively. The highest infection rate of *Cyclospora* spp. was found in Holstein cattle aged 6–12 months, whereas the lowest infection rate was found in 0–3 months. A Chi-squared test showed that the prevalence of *Cyclospora* spp. in four age groups showed no significant difference (*p* > 0.05) (Table 1). In total, 102 fecal samples from male cows and 422 samples from female cows were collected from Dali, Kunming, Qujing, and Chuxiong. Comparing the infection in different sexes, we found that the infection rate in females and males was 2.37% and 2.94%, respectively. There was no significant difference between the sexes (*p* > 0.05) (Table 1).

### 3.3. Phylogenetic Analyses

**Figure 2:**
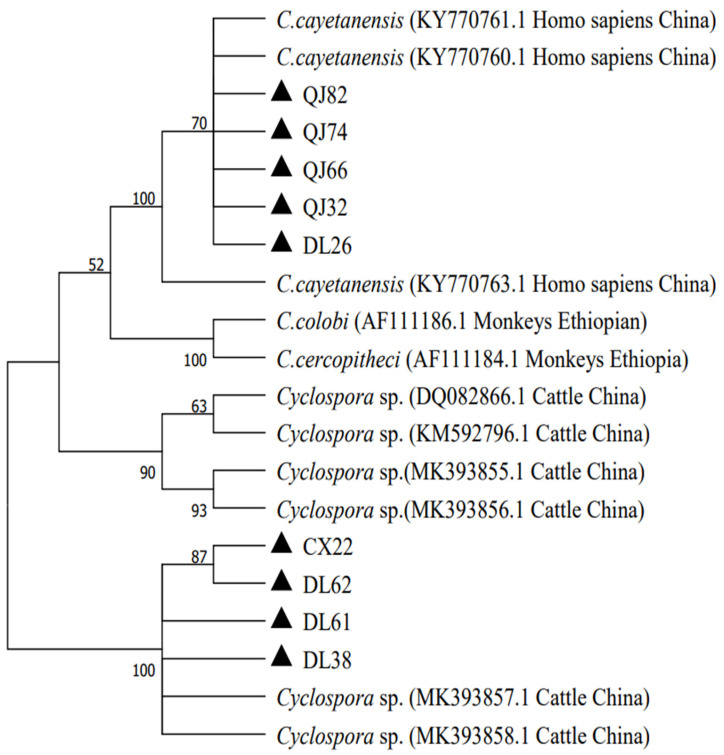
Phylogenetic relationship of *Cyclospora* spp. of Holstein cattle in the Yunnan Province. The genotypes identified in this study are marked with ▲.

The PCR products that were successfully digested by the restriction enzyme *Bsp E* I were bidirectionally sequenced. In the present study, a total of 13 positive samples were successfully sequenced. The sequences of five PCR amplicons showed 99% similarities to the *C. cayetanensis* sequence in the GenBank database, and the other sequences were more similar to the *Cyclospora* spp. of the cattle. In the present study, we selected 4, 4, and 1 representative sequences from Dali, Qujing, and Chuxiong to construct phylogenetic relationships. In this study, the *Cyclospora* spp. were mainly divided into two branches. Four samples from Qujing and one sample from Dali were co-located with *C. cayetanesis* (KY 7707760) and formed a monophyletic group in the tree, while the other samples formed a separate clade with the *Cyclospora* spp. of the *Bos frontalis* in the Yunnan Province. The phylogenetic analysis result of genetic evolution analysis is shown in Figure 2.

## 4. Discussion

The traditional method for the detection of *Cyclospora*-like oocysts in animal fecal specimens is through microscopic observation [21]. The morphologies among *Eimeria* spp., *Cyclospora* spp., and *Cryptosporidium* spp. are similar. However, *Cyclospora* spp. and *Cryptosporidium* spp. can be distinguished from the size of oocysts and sporulation status in fresh feces. The *Cyclospora* spp. in fresh feces is not sporulated and is 8–10 μm in diameter, while *Cryptosporidium* spp. is sporulated and 4–6 μm in diameter. Additionally, modified Ziehl–Neelsen staining can also be used for distinguishing *Cryptosporidium* from *Cyclospora* [22]. Unfortunately, the morphology characters of *Cyclospora* spp. are far more similar to *Eimeria* spp. than those of *Cryptosporidium* spp. [4], and we can not distinguish *Cyclospora* spp. from *Eimeria* spp. by using microscopic examination. The SSU rRNA gene is highly conserved, and it has been a good genetic marker for distinguishing *Cyclospora* spp. from other apicomplexan protozoa [23,24]. However, previous studies showed that the primers used for amplifying the small subunit SSU rRNA genes of *Cyclospora* spp. can also amplify the small subunit SSU rRNAs of *Eimeria* spp. [23,25]. Restriction fragment length polymorphism (RFLP) analysis was then developed based on the nucleotide differences in the amplified region [26], and it has been a powerful tool in the classification and identification of parasite species, parasite epidemiology research, and genetic differentiation among closely related species. In 1995, Relman et al. [23] used restriction enzyme *Mn II* to distinguish *Cyclospora* spp. from *Eimeria* spp. In 1998, Jinneman KC et al. used the restriction endonuclease *Mn II* to distinguish *Cyclospora* spp. from *Eimeria* spp. [26]. Subsequently, a new PCR- RFLP was developed for ruminants. The restriction endonuclease *Kpn I* could distinguish the *Cyclospora* species from *Eimeria* species in ruminants, and the PCR amplicons of *Eimeria* spp. can be digested into 80 and 420 bp [20]. However, most of the time, 80 bp can not be clearly observed, and there are certain requirements for marker, agarose, and recognition ability. In this study, the restriction endonuclease *Bsp E* I was used for enzymatic cleavage, and the positive band of *Cyclospora* spp. was successfully cleaved into two parts 130 bp and 370 bp, while *Eimeria* spp. was not. The results of the present study indicate that PCR- RFLP, which was based on *Bsp E* I, can be an alternative method to distinguish *Cyclospora* spp. from *Eimeria* spp. of cattle. Whether this method can distinguish *Cyclospora* spp. from *Eimeria* spp. of the other animals remains to be determined.

In China, the research on *Cyclospora* spp. mainly focuses on human beings, non-human primates, crops and water sources. In 2002, Wang et al. [15] examined the feces of people in the Anhui province and found that the infection rate of *C. cayetanensis* in the Anhui province was 2.3%. Zhang et al. examined the fresh feces from diarrhea cases in 7 counties/cities in the Yunnan Province and found that infection rate of *C. cayetanensis* in diarrhea cases in the Yunnan Province was 3.97% [27]. In 2011, Zhou et al. studied the infection of *C. cayetanensis* in hospitalized patients in Henan and found that the prevalence rate ratio was 0.70% [16]. At present, *Cyclospora* spp. infection has been detected in non-human primates such as *Macaca fascicularis*, the primate *Rhinopithecus roxellanae*, and rhesus monkeys (*Macaca mulatta*) in China, and the prevalence of *Cyclospora* spp. in non-human primates ranged from 5.56 to 10.52% [11,19,28]. In addition, *Cyclospora* spp. was also detected in vegetables, fruits, and water in China. Li et al. found that the positive rate of *Cyclospora* spp. on the surface of vegetables and fruits in Henan province was 0.2% [29]. Fang et al. found that the *Cyclospora* contamination rate in wastewater treatment plants and sewer samples in Guangzhou city was 0.42% and 3.41%, respectively [30]. However, there are few studies on the *Cyclospora* spp. of bovine in China, and information on *Cyclospora* spp. infection in Holstein cattle in Yunnan is lacking. We studied the infection of *Cyclospora* spp. in Holstein cattle in Yunnan by performing molecular biology tests on fecal samples of Holstein cattle and genetic evolution analyses.

The total positive rate of *Cyclospora* spp. in Holstein cattle in the Yunnan Province was 2.48%, and the infection of *Cyclospora* spp. was detected in Dali, Chuxiong, and Qujing. The infection rates among Dali, Chuxiong, and Qujing were not significant (*p* > 0.05), suggesting that *Cyclospora* spp. infections were common in Yunnan and that regional factors did not show significant influences on the infection statuses of *Cyclospora* spp. Interestingly, *Cyclospora* spp. infection was not found in Kunming, which may be due to better feeding and environment management in Kunming. Basnett K examined the feces of Indian sheep for studying the infection of *Cyclospora* spp. and found a 2.14% prevalence of *Cyclospora* spp. infection in small ruminants [31], which was similar to the results of the present study. In this study, the rate of *Cyclospora* spp. infection at different ages ranged from 1.74% to 9.09%, and it was found that the rate of *Cyclospora* spp. infection gradually increased with age, the highest rate of *Cyclospora* spp. infection was found in Holstein cattle at 6–12 months of age, and the infection rate of *Cyclospora* spp. gradually decreased as age increased. However, the difference in the rates of *Cyclospora* spp. infection among different ages was not significant (*p* > 0.05).

In this study, the infection rate in male was 2.94% and the infection rate in female was 2.37%, and the difference in the infection rate of *Cyclospora* spp. between the sexes was not significant (*p* > 0.05), which indicated that the factor of sex had no significant effect on the infection of. *Cyclospora* spp. in Holstein cattle. Previous studies have suggested that cyclosporiasis is associated with environmental factors, such as season and local climate [7,32,33,34]. This could be due to the fact that *Cyclospora* oocysts need a sporulation process to become infective, and the oocyst sporulation rate depends on various environmental factors, such as temperature and humidity. Both a room temperature and humid environment is suited for the sporulation of *Cyclospora* oocysts to form infectious oocysts [35,36,37]. Thus, the infection of *Cyclospora* spp. is mostly seasonal, and the infection of *Cyclospora* spp. will increase significantly in the rainy season or summer [7,32,33,34,38,39,40].

An analysis of the 13 sequences of *Cyclospora* spp. found in this study revealed that five of them were closely related to *C. cayetanensis* samples isolated from humans in the Henan province (with a 99% similarity), whereas the remaining eight sequences were closely related to the *Cyclospora* spp. of bovines isolated from Guangzhou, Yunnan, and Henan. *Cyclospora* spp. can be spread through fruits and vegetables, as well as through water and soil, posing a threat to human life and health [13,36]. Current studies suggest that *C. cayetanensis* is the only known *Cyclospora* spp. that can infect humans [2,11]. Additionally, *C. cayetanensis* can be detected in the feces of other animals. The presence of *C. cayetanensis* oocysts in the feces of animals does not necessarily indicate infection. It may be due to the fact that animals can act as paratenic hosts, which can aid in the dissemination of oocysts into the environment [4,36].

Domestic animals are both reservoirs and sources of infection for zoonotic pathogens, and they are in frequent contact with breeders closely related to human activities. Infections with *Cyclospora* spp. could be a public health hazard and a potential threat to human health. Previous studies have detected the presence of *Cyclospora* spp. in Yunnan, but there has no investigation into the infection of *Cyclospora* spp. in Holstein cattle in Yunnan. In this study, *Cyclospora* spp. was detected in the feces of Holstein cattle, which indicated that there was a Cyclospora spp. infection in Holstein cattle in Yunnan. Most importantly, some of the bovine-derived *Cyclospora* spp. were closely related to human *Cyclospora* spp. This suggested that there is a potential zoonotic risk for *Cyclospora* spp. spread via the feces of Holstein cattle in Yunnan. At present, the risk of human infection due to the animal transmission of *Cyclospora* oocysts has not yet been evaluated [8]. Thus, the use of RFLP coupled with DNA sequencing to confirm the species of *Cyclospora* spp. in animals is of great importance for public health safety.

## 5. Conclusions

This is the first study to reveal the prevalence of *Cyclospora* spp. in Holstein cattle in the Yunnan Province, China (2.48%, 13/524). The infection of *Cyclospora* spp. is not statistically significant in different regions, sexes, and ages. In this study, our phylogenetic analysis showed that five samples were closely associated with *C. cayetanensis* in humans and located on the same branch with the *C. cayetanensis* zoonotic group, which indicated that the *Cyclospora* spp. infection in Holstein cattle in the Yunnan Province had the risk of zoonosis. It is necessary to strengthen the prevention and control of *Cyclospora* spp.

## Figures and Tables

**Table 1:** The infection of *Cyclospora* spp. in Holstein cattle in partial areas of the Yunnan Province.

Factors	Category	No. Tested	No. Positive	Infection Rate (%) (95%CI)	OR (95%, CI)	*p*-Value
Region	Dali	274	6	2.19 [0.45–3.93]	Reference	0.339
	Kunmin	57	0	0 [0.00–0.00]	-	
	Qujing	158	5	3.16 [0.41–5.92]	1.46 (0.44–4.86)	
	Chuxiong	35	2	5.71 [0.00–13.80]	2.71 (0.53–13.96)	
Age (M)	0–3	344	6	1.74 [0.35–3.13]	Reference	0.204
	3–6	57	3	5.26 [0.00–11.24]	3.13 (0.76–12.89)	
	6–12	11	1	9.09 [0.00–29.35]	5.63 (0.62–51.27)	
	>12	112	3	2.68 [0.00–5.72]	1.55 (0.38–6.30)	
Sex	Female	422	10	2.37 [0.91–3.82]	Reference	0.739
	Male	102	3	2.94 [0.00–6.28]	1.25 (0.34–4.62)	
Total		524	13	2.48	-	-

No.: number; CI: confidence interval; OR: odds ratio.

## Data Availability

The data that support the findings are in the possession of the authors. The data of this study are available on request from the corresponding author.

## Supplementary Materials

The following supporting information can be downloaded at: https://www.mdpi.com/article/10.3390/ani13091527/s1, Figure S1: Original Western Blot Figure.

## References

[1] Shields J.M., Olson B.H. (2003). *Cyclospora**cayetanensis*: A review of an emerging parasitic coccidian. Int. J. Parasitol..

[2] Lainson R. (2005). The genus *Cyclospora* (Apicomplexa: Eimeriidae), with a description of *Cyclospora schneideri* n.sp. in the snake Anilius scytale scytale (Aniliidae) from Amazonian Brazil—A review. Mem. Inst. Oswaldo Cruz.

[3] Insulander M., Svenungsson B., Lebbad M., Karlsson L., de Jong B. (2010). A foodborne outbreak of *Cyclospora* infection in Stockholm, Sweden. Foodborne Pathog. Dis..

[4] Ortega Y.R., Sanchez R. (2010). Update on *Cyclospora cayetanensis*, a food-borne and waterborne parasite. Clin. Microbiol. Rev..

[5] Ortega Y.R., Nagle R., Gilman R.H., Watanabe J., Miyagui J., Quispe H., Kanagusuku P., Roxas C., Sterling C.R. (1997). Pathologic and clinical findings in patients with cyclosporiasis and a description of intracellular parasite life-cycle stages. J. Infect. Dis..

[6] Hart A.S., Ridinger M.T., Soundarajan R., Peters C.S., Swiatlo A.L., Kocka F.E. (1990). Novel organism associated with chronic diarrhoea in AIDS. Lancet.

[7] Chacin-Bonilla L. (2008). Transmission of *Cyclospora cayetanensis* infection: A review focusing on soil-borne cyclosporiasis. Trans. R Soc. Trop. Med. Hyg..

[8] Totton S.C., O’Connor A.M., Naganathan T., Martinez B.A.F., Sargeant J.M. (2021). A review of *Cyclospora cayetanensis* in animals. Zoonoses Public Health.

[9] Long E.G., White E.H., Carmichael W.W., Quinlisk P.M., Raja R., Swisher B.L., Daugharty H., Cohen M.T. (1991). Morphologic and staining characteristics of a cyanobacterium-like organism associated with diarrhea. J. Infect. Dis..

[10] Onstad N.H., Miller M.R., Green M.L., Witola W.H., Davidson P.C. (2019). A Review of *Cyclospora cayetanensis* Transport in the Environment. Trans. ASABE.

[11] Li N., Ye J., Arrowood M.J., Ma J., Wang L., Xu H., Feng Y., Xiao L. (2015). Identification and morphologic and molecular characterization of *Cyclospora macacae* n. sp. from rhesus monkeys in China. Parasitol. Res..

[12] Dubey J.P., Khan A., Rosenthal B.M. (2022). Life Cycle and Transmission of *Cyclospora cayetanensis*: Knowns and Unknowns. Microorganisms.

[13] Onstad N.H., Beever J.E., Miller M.R., Green M.L., Witola W.H., Davidson P.C. (2019). *Cyclospora**Cayetanensis* Presence in the Environment—A Case Study in the Chicago Metropolitan Area. Environments.

[14] Chu D.M., Sherchand J.B., Cross J.H., Orlandi P.A. (2004). Detection of *Cyclospora cayetanensis* in animal fecal isolates from Nepal using an FTA filter-base polymerase chain reaction method. Am. J. Trop. Med. Hyg..

[15] Wang K.X., Li C.P., Wang J., Tian Y. (2002). *Cyclospore cayetanensis* in Anhui, China. World J. Gastroenterol..

[16] Zhou Y., Lv B., Wang Q., Wang R., Jian F., Zhang L., Ning C., Fu K., Wang Y., Qi M. (2011). Prevalence and molecular characterization of *Cyclospora cayetanensis*, Henan, China. Emerg. Infect. Dis..

[17] Eberhard M.L., da Silva A.J., Lilley B.G., Pieniazek N.J. (1999). Morphologic and molecular characterization of new *Cyclospora* species from Ethiopian monkeys: *C. cercopitheci* sp.n., *C. colobi* sp.n., and *C. papionis* sp.n. Emerg. Infect. Dis..

[18] Lopez F.A., Manglicmot J., Schmidt T.M., Yeh C., Smith H.V., Relman D.A. (1999). Molecular characterization of *Cyclospora*-like organisms from baboons. J. Infect. Dis..

[19] Zhao G.H., Cong M.M., Bian Q.Q., Cheng W.Y., Wang R.J., Qi M., Zhang L.X., Lin Q., Zhu X.Q. (2013). Molecular characterization of *Cyclospora*-like organisms from golden snub-nosed monkeys in Qinling Mountain in Shaanxi province, northwestern China. PLoS ONE.

[20] Li G., Xiao S., Zhou R., Li W., Wadeh H. (2007). Molecular characterization of *Cyclospora*-like organism from dairy cattle. Parasitol. Res..

[21] Eberhard M.L., Pieniazek N.J., Arrowood M.J. (1997). Laboratory diagnosis of *Cyclospora* infections. Arch. Pathol. Lab. Med..

[22] Khanna V., Tilak K., Ghosh A., Mukhopadhyay C. (2014). Modified negative staining of heine for fast and inexpensive screening of *Cryptosporidium*, *Cyclospora*, and *Cystoisospora *spp.. Int. Sch. Res. Not..

[23] Relman D.A., Schmidt T.M., Gajadhar A., Sogin M., Cross J., Yoder K., Sethabutr O., Echeverria P. (1996). Molecular phylogenetic analysis of *Cyclospora*, the human intestinal pathogen, suggests that it is closely related to *Eimeria* species. J. Infect. Dis..

[24] Sulaiman I.M., Ortega Y., Simpson S., Kerdahi K. (2014). Genetic characterization of human-pathogenic *Cyclospora cayetanensis* parasites from three endemic regions at the 18S ribosomal RNA locus. Infect. Genet. Evol..

[25] Pieniazek N.J., Slemenda S.B., da Silva A.J., Alfano E.M., Arrowood M.J. (1996). PCR confirmation of infection with *Cyclospora cayetanensis*. Emerg. Infect. Dis..

[26] Jinneman K.C., Wetherington J.H., Hill W.E., Adams A.M., Johnson J.M., Tenge B.J., Dang N.L., Manger R.L., Wekell M.M. (1998). Template preparation for PCR and RFLP of amplification products for the detection and identification of *Cyclospora* sp. and *Eimeria* spp. Oocysts directly from raspberries. J. Food Prot..

[27] Zhang B.X., Yu H., Zhang L.L., Tao H., Li Y.Z., Li Y., Cao Z.K., Bai Z.M., He Y.Q. (2002). Prevalence survey on *Cyclospora cayetanensis* and *Cryptosporidium* ssp. in diarrhea cases in Yunnan Province. Zhongguo Ji Sheng Chong Xue Yu Ji Sheng Chong Bing Za Zhi.

[28] Ye J., Xiao L., Li J., Huang W., Amer S.E., Guo Y., Roellig D., Feng Y. (2014). Occurrence of human-pathogenic *Enterocytozoon bieneusi*, *Giardia duodenalis* and *Cryptosporidium* genotypes in laboratory macaques in Guangxi, China. Parasitol. Int..

[29] Li J., Shi K., Sun F., Li T., Wang R., Zhang S., Jian F., Ning C., Zhang L. (2019). Identification of human pathogenic *Enterocytozoon bieneusi*, *Cyclospora cayetanensis*, and *Cryptosporidium parvum* on the surfaces of vegetables and fruits in Henan, China. Int. J. Food Microbiol..

[30] Fan Y., Wang X., Yang R., Zhao W., Li N., Guo Y., Xiao L., Feng Y. (2021). Molecular characterization of the waterborne pathogens *Cryptosporidium* spp., *Giardia duodenalis*, *Enterocytozoon bieneusi*, *Cyclospora cayetanensis* and *Eimeria* spp. in wastewater and sewage in Guangzhou, China. Parasit. Vectors.

[31] Basnett K., Nagarajan K., Soundararajan C., Vairamuthu S., Rao G.V.S. (2018). Morphological and molecular identification of *Cyclospora* species in sheep and goat at Tamil Nadu, India. J. Parasit. Dis..

[32] Helmy M.M. (2010). *Cyclospora cayetanensis*: A review, focusing on some of the remaining questions about cyclosporiasis. Infect. Disord. Drug. Targets.

[33] Li J., Wang R., Chen Y., Xiao L., Zhang L. (2020). *Cyclospora cayetanensis* infection in humans: Biological characteristics, clinical features, epidemiology, detection method and treatment. Parasitology.

[34] Frickmann H., Alker J., Hansen J., Dib J.C., Aristizabal A., Concha G., Schotte U., Kann S. (2021). Seasonal Differences in *Cyclospora cayetanensis* Prevalence in Colombian Indigenous People. Microorganisms.

[35] Ortega Y.R., Sterling C.R., Gilman R.H. (1998). *Cyclospora* *cayetanensis*. Adv. Parasitol..

[36] Almeria S., Cinar H.N., Dubey J.P. (2019). *Cyclospora cayetanensis* and Cyclosporiasis: An Update. Microorganisms.

[37] Connor B.A. (1997). *Cyclospora* infection: A review. Ann. Acad. Med. Singap..

[38] Hall R.L., Jones J.L., Hurd S., Smith G., Mahon B.E., Herwaldt B.L. (2012). Population-based active surveillance for *Cyclospora* infection--United States, Foodborne Diseases Active Surveillance Network (FoodNet), 1997–2009. Clin. Infect. Dis..

[39] Kaminsky R.G., Lagos J., Santos G.R., Urrutia S. (2016). Marked seasonality of *Cyclospora cayetanensis* infections: Ten-year observation of hospital cases, Honduras. BMC Infect. Dis..

[40] Jiang Y., Yuan Z., Zang G., Li D., Wang Y., Zhang Y., Liu H., Cao J., Shen Y. (2018). *Cyclospora cayetanensis* infections among diarrheal outpatients in Shanghai: A retrospective case study. Front. Med..

