# Supplementary figures and images for: Molecular Epidemiological Investigation of Cyclospora spp. in Holstein Cattle in Partial Areas of the Yunnan Province, China
Source: Animals (Basel). 2023 May 3;13(9):1527. doi: 10.3390/ani13091527 (PMC10177582; doi:10.3390/ani13091527)

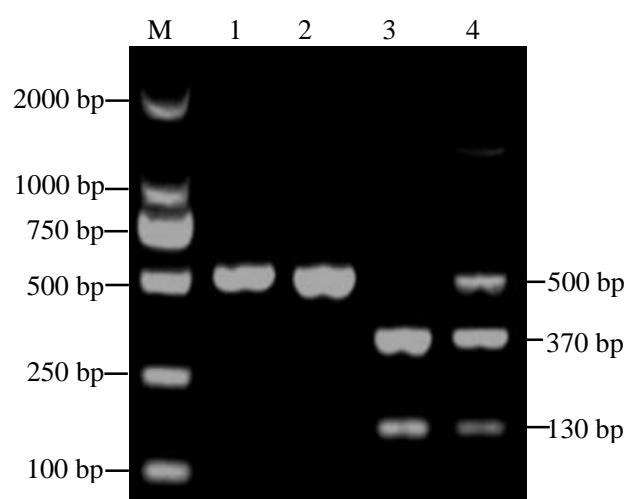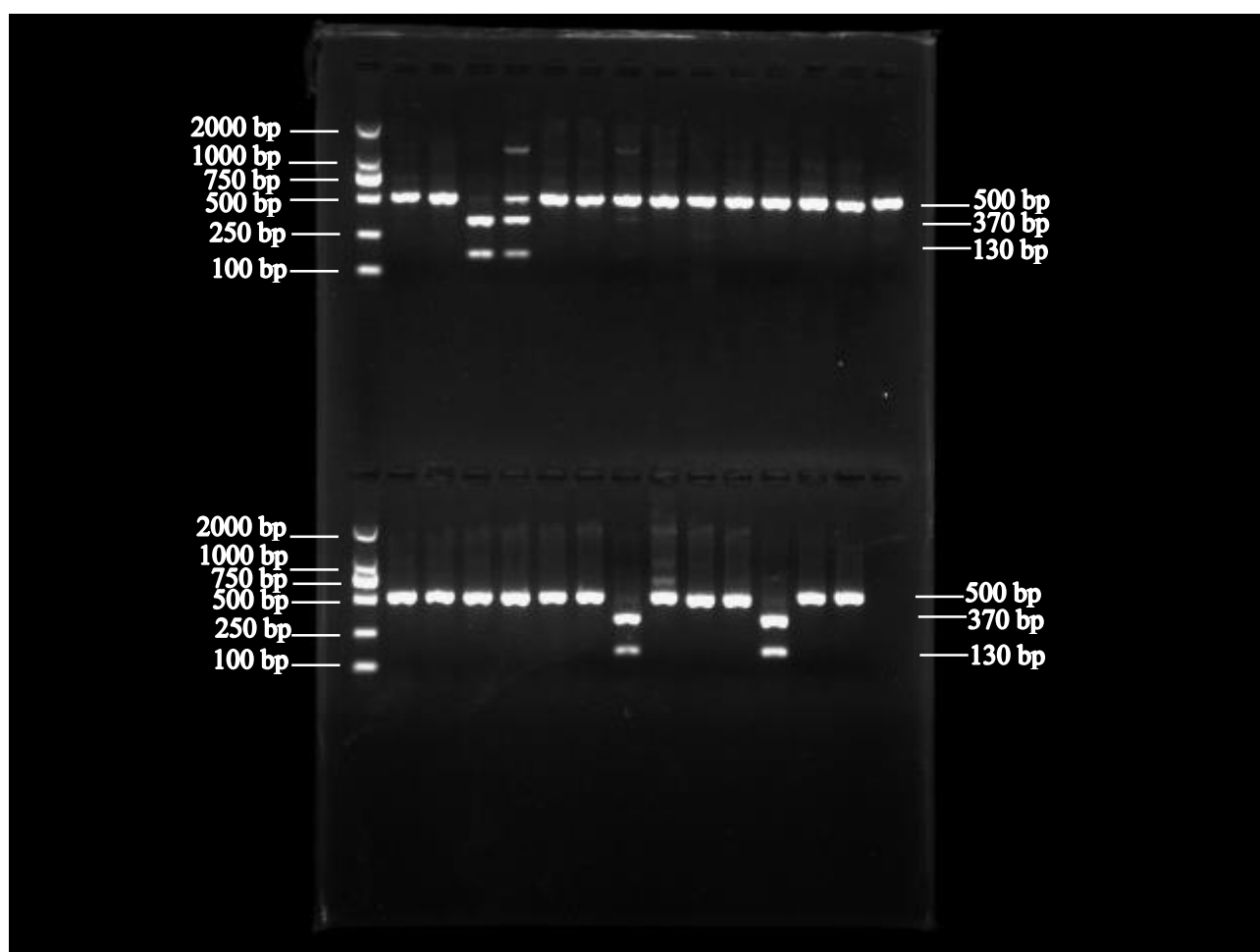

**Figure S1.** Original Western Blot Figure.

Supplement: Supplementary file 1 [file animals-13-01527-s001.zip › animals-2309922-supplementary.pdf]
